# Practices and barriers to screening for hyperglycaemia in pregnancy among providers of antenatal care in Jos, Nigeria

**DOI:** 10.4102/ajlm.v11i1.1845

**Published:** 2022-10-31

**Authors:** Lucius C. Imoh, Abdulazis S. Longwap, Favour E. Haruna, Oghale J. Asieba, Joy P. Istifanus, Joy A. Imoh, Mathilda E. Banwat

**Affiliations:** 1 Department of Chemical Pathology, Faculty of Medical Sciences, University of Jos, Jos, Plateau State, Nigeria; 2 Department of Geography and Planning, Faculty of Environmental Science, University of Jos, Jos, Plateau State, Nigeria; 3 Department of Community Medicine, Faculty of Medical Sciences, University of Jos, Jos, Plateau State, Nigeria

**Keywords:** hyperglycaemia in pregnancy, gestational diabetes mellitus, guidelines for gestational diabetes mellitus, screening practices, oral glucose tolerance test, low middle-income countries

## Abstract

**Background:**

Screening for hyperglycaemia in pregnancy (HIP) is an important component of comprehensive antenatal care. Screening practices for HIP in Nigeria and factors that influence these practices are not well understood.

**Objective:**

We examined the screening practices for HIP and their correlates among antenatal healthcare providers (AHPs).

**Methods:**

This descriptive cross-sectional study of AHPs providing all levels of antenatal care was conducted between August 2019 and September 2019 in Jos, Nigeria. Eligible AHPs completed a semi-structured, self-administered questionnaire, and data were analysed for adherence to recommended screening practices such as World Health Organization, International Association of Diabetes and Pregnancy Study Groups and National Institute for Health and Care Excellence guidelines.

**Results:**

Of the 128 respondents included in the analysis, 59 (46.1%) were male and 69 (53.9%) were female. The mean participant age was 35.7 years (standard deviation: ± 8.5 years). Most (68.0%) screened all pregnant women (universal screening) for gestational diabetes mellitus. Fasting blood glucose (77.0%) and random blood glucose (55.7%) were the most common tests used. Only 27 respondents (22.1%) screened using the 75 g oral glucose tolerance test, and most were doctors, AHPs in faith-based or government institutions, tertiary institutions and facilities with availability of automated glucose analysers (*p* < 0.05 for all).

**Conclusion:**

Screening practices for HIP among the AHPs do not generally conform to best practices. Hence, there is an urgent need for implementation of universal guidelines and provision of regular updates and basic glucose measuring devices for AHPs at all healthcare levels.

## Introduction

The incidence of gestational diabetes mellitus (GDM) and overt diabetes in pregnancy are rising globally in tandem with rising trends of obesity, diabetes mellitus (DM) and metabolic syndrome. The epidemiological transition, which is associated with rise in incidence of non-communicable diseases in low- and middle-income countries, suggests that diseases like DM will increasingly become more prevalent in Nigeria.^1^ Indeed, the greatest rise in noncommunicable diseases and deaths due to noncommunicable diseases is predicted to occur in developing and poor-resource countries like Nigeria.^2,3^ Recent studies have demonstrated an increase over time in the prevalence of GDM in Nigeria.^4^ Remarkably, the prevalence of GDM in Jos, Plateau state, in recent studies has been shown to be comparable with prevalence in regions of the world with a high burden of DM.^5,6^

Hyperglycaemia in pregnancy (HIP) has the potential to worsen maternal and child health indices, thus making it of public health concern. The International Federation of Gynecology and Obstetrics recommends that HIP and GDM be considered a global health priority.^7,8^ The International Federation of Gynecology and Obstetrics advocates for prevention, screening, early diagnosis and management of HIP as an important intervention towards achieving Sustainable Development Goal 3, which partly focuses on reducing maternal and child mortalities.^9,10^

The modalities for screening and diagnosis of diabetes outside of pregnancy are universally accepted. However, the same cannot be said for the pregnant population. Although stakeholders agree on the need for diagnosing and managing HIP, contentious issues regarding screening for diabetes in pregnancy include: the type of test to use for screening, the criteria for diagnosis, whom to screen (universal vs risk-based or selective screening) and when to screen, among others.^9,10^ These can be influenced by several factors including age and experience of the healthcare worker, cadre of antenatal healthcare provider (AHP), specialty of AHP, socio-economic determinants like cost of screening or availability of laboratory facilities and administrative or organisational factors such as availability of policies, guidelines and standard operating procedures.^11,12,13^ There appears to be no universally acceptable guideline for screening in Nigeria, as the modalities for screening are left to the discretion of the health provider.

The disparity in knowledge and screening practices across health centres in Nigeria has implications for early diagnosis, referral and management of women with hyperglycaemia. First-line healthcare providers, particularly nurses and midwives, are usually the first point of contact for pregnant women. The screening practices they adopt may make a significant difference in identifying pregnant women with potential adverse outcomes. The demand to streamline the screening diagnosis and management of HIP in Nigeria has to begin with examination of the current practices and their determinants. Unfortunately, there is a paucity of data on the subject of screening practices for HIP in Nigeria. It is therefore important to assess the current screening practices for HIP by first-line AHPs in healthcare facilities. Most studies on GDM and DM in pregnancy have focused on women accessing care in tertiary and academic healthcare centres. Not much is known regarding practices in private or rural settings. This study hopes to evaluate the practices concerning screening, diagnosis and management of HIP in different cadres of healthcare centres in Jos, Nigeria, with a view to understand the underlying determinants that may be associated with them and their implication for maternal health.

## Methods

### Ethical considerations

This study was carried out after due approval from the Human Research Ethical Committee of the Jos University Teaching Hospital (DCS/ADM/127/XXIX/1692). Written permission was obtained from the Plateau State Ministry of Health. Appropriate permission was obtained from the relevant authorities of the health facilities. Written informed consent was obtained from all participants after due explanation of the research work and procedures. Anonymity and confidentiality of the information obtained from the participants in this study was assured and maintained.

### Study design

This was a cross-sectional descriptive study conducted between August 2019 and September 2019 to survey the screening practices and their correlates among AHPs.

### Study area

This study was conducted in Jos North Local Government Area (LGA) in Plateau state. Plateau state is one of the 36 states in Nigeria located in the North Central geographical zone. Jos North is one of the LGAs of Jos, the capital city of Plateau state with a cosmopolitan nature, inhabited by people from all tribes in Nigeria. Antenatal care is provided at primary, secondary and tertiary healthcare facilities and accessed by persons in the entire Plateau state and also people living in the neighbouring states in the North Central and North East regions of Nigeria.

### Study participants and selection criteria

This study was conducted among AHPs of different cadres at all levels of primary, secondary and tertiary healthcare centres in Jos North LGA. This included doctors, nurses and community health providers such as community health extension workers and community health officers, and front-line staff directly involved in providing antenatal care in primary, secondary and tertiary healthcare centres in Jos North LGA who gave consent to participate in the study. Any AHPs on leave or unavoidably absent during the period of the study or who had worked < 3 months at the current health facility were not included in the study.

The sample size was determined by the formula for cross-sectional study and finite population correction:^14^


$$n=\frac{Z^{2}pq}{\partial^{2}}$$ [Eqn 1]

In Equation 1, *n* is the desired sample size, *Z* is the standard normal deviation corresponding to a 95% level of confidence. The value obtained from a standard normal distribution is 1.96. *p* is the prevalence of screening for HIP. Using an assumed prevalence of screening for HIP of 50%:


$$\begin{array}{l} \text{Sample size }(n)=\frac{{\left(1.96\right)}^{2}\times 0.5(1-0.5)}{0.05\times 0.05}\\ \;=\frac{3.84\times 0.5\times 0.5}{0.0025}\\ \;=384 \end{array}$$ [Eqn 2]

Further correction of the sample size was done, because the population of AHPs in Jos North LGA is less than 10 000. The formula for this correction is:


$$\text{nf}=\frac{n}{1+\left(\frac{n}{N}\right)}$$ [Eqn 3]

In Equation 3, nf is the desired sample size when the population is less than 10 000. *n* is the desired sample size when the population is more than 10 000 = 384 and *N* is the population size of AHPs in Jos North LGA = 140. Therefore:


$$\text{nf}=\frac{4}{1+\frac{384}{140}}=140\text{ AHPs}$$ [Eqn 4]

A value of 10% of the minimum sample size was added to the study to account for non-response and incomplete data, bringing the total sample size to 115 AHPs.

### Sampling technique

A multi-stage sampling technique was employed. There are 69 health facilities (private and public; primary, secondary and tertiary) that provide antenatal care services in Jos North LGA according to data from the State Ministry of Health. All the health facilities and AHPs who gave consent were included in the study. Potential participants were provided with a semi-structured questionnaire to complete at their convenience.

### Data collection instrument

Data were collected using a pretested self-administered structured questionnaire organised in four parts (part A: socio-demographics; B: screening practice and diagnosis of HIP; C: diagnostic criteria for HIP; D: constraints to screening for HIP). The questionnaire was developed following extensive review of the available literature on recommended screening practices such as World Health Organization, International Association of Diabetes and Pregnancy Study Groups and National Institute for Health and Care Excellence guidelines.^10^ It was written in English, given the high level of English fluency among the target participants. The participants were allowed to select all options that applied to a given question and the freedom to select ‘Not sure’ if they were uncertain of their response. For questions related to challenges and constraints to adequate screening and diagnosis for GDM and DM in pregnancy, participants responded on a five-point Likert scale: ‘Strongly agree’, ‘Agree’, ‘Neutral’, ‘Disagree’ and ‘Strongly disagree’.

### Procedure for data collection

At each selected health facility, each eligible respondent was given a detailed explanation of the research by the researcher or trained research assistants. After obtaining informed consent the questionnaire was distributed to the participants who self‑administered them independently and returned them to the researcher on completion. The researcher and trained research assistants served as a supervisor to ensure data quality by checking the completeness of questionnaires.

### Data analysis

Data were cleaned and entered into Microscoft Excel^®^ version 15.0 (Microsoft Corp. 2013, Redmond, Washington, United States) and exported to Statistical Product and Service Solutions version 23.0 (IBM Corp. 2015, Armonk, New York, United States) software for statistical analysis. Descriptive statistics were presented as mean values ± standard deviation or medians with interquartile ranges for non-normal continuous variables, and proportions (as percentages) for categorical variables. Tables and graphical representations were used to summarise the data. Statistical associations of dependent and independent variables were assessed using Chi-square tests or Yate’s correction test for continuity where the Chi-square test would not be appropriate. All tests were two-tailed, a 95% confidence interval was used and *p*-values < 0.05 were considered statistically significant.

## Results

A total of 193 questionnaires were returned by AHPs in 60 health facilities. Of these, only 159 respondents had worked for at least three months in the current health facility and 128 (80.5%) responded that they screen for GDM and were selected for further analysis.

### Socio-demographic characteristics of the study participants

Fifty-nine (46.1%) of the respondents were male and 69 (53.9%) were female healthcare workers (Table 1). The ages of the participants ranged from 21 to 60 years with a mean of 35.6 ± 8.2 years. Most (47.6%, 61/128) of the respondents worked in private health facilities and 44.5% (57/128) provided primary healthcare. Forty-four (34.4%) of the participants were doctors, and obstetrics and gynaecology (76.5%, 13/17) was the most common specialty among doctors with clinical specialties.

**TABLE 1:** General characteristics of antenatal healthcare providers in healthcare facilities in Jos North, Plateau state, Nigeria, August to September 2019.

Variable	Frequency	Percentage
**Gender (*n* = 128)**		
Male	59	46.1
Female	69	53.9
**Institution category (*n* = 128)**		
Faith-based	12	9.4
Private	61	47.6
Government or public	55	43.0
**Level of institution (*n* = 128)**		
Primary	57	44.5
Secondary	25	19.5
Tertiary	46	36.0
**Job designation (*n* = 128)**		
Doctor	44	34.4
Nurse or midwife	36	28.1
Community health officer	9	7.0
Community health extension worker	30	23.4
Non-specified	9	7.0
**Cadre of doctors (*n* = 44)**		
House officer	7	15.9
Medical officer	11	25.0
Registrar	4	9.1
Senior Registrar	7	15.9
Consultant	11	25.0
Non-specified	4	9.1
**Doctor’s specialty (*n* = 17)**		
General practice	4	23.5
Obstetrics and gynaecology	13	76.5

Note: Age: mean = 35.6; standard deviation = 8.2.

### Screening practices of respondents

The most common period for screening was the second trimester (75.0%; 96/128) (Table 2). Most (68.0%; 87/128) of the respondents screened all pregnant women for GDM (universal screening). The majority (49.2%; 63/128) of the respondents practised a laboratory screening method only, while (46.1%; 59/128) used a combination of in-clinic and laboratory methods.

**TABLE 2:** Summary of screening practices and challenges according to antenatal healthcare providers in healthcare facilities in Jos North, Plateau state, Nigeria, August to September 2019.

Screening practice	Frequency†	Percentage
**When screening is done (*n* = 128)**		
First trimester	88	68.8
Second trimester	96	75.0
Third trimester	82	64.1
**Who is screened (*n* = 128)**		
All pregnant women (universal screening)	87	68.0
Pregnant women at risk (risk-based screening)	31	24.2
No response	10	7.8
**Screening method (*n* = 128)**		
In-clinic only	3	2.3
Laboratory only	63	49.2
In-clinic and laboratory	59	46.1
No response	3	2.3
**Methods for glucose testing (*n* = 122)**		
Glucometer	108	88.5
Dipstick urine glucose	100	82.0
Automated chemistry analysers	16	13.1
Manual glucose assays	2	1.6
Send-out test	20	16.4
**Challenges to screening and diagnosis of hyperglycaemia in pregnancy (*n* = 122)** ‡		
Late booking	104	85.2
Lack of guidelines	92	75.4
Lack of information or inadequate knowledge	78	63.9
Lack of qualified personnel for testing	57	46.7
Lack of testing equipment	53	43.4
High cost of testing	35	28.7
Discomfort of testing	34	27.9
Delay in obtaining results	27	22.1

† , Respondents selected all that apply; hence, more than one response per respondents may be allowed as applicable in practice;

‡ , *n* = 122 (Among those who screened by laboratory methods).

### Risk factors and prompting for screening

**FIGURE 1:**
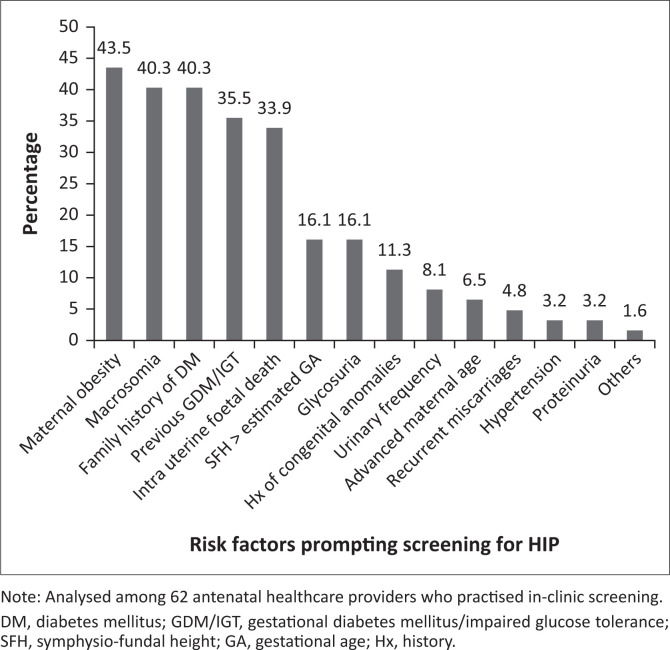
Risk factors prompting screening for hyperglycaemia in pregnancy among antenatal healthcare providers in healthcare facilities in Jos North, Plateau state, Nigeria, August to September 2019.

Among those who practised in-clinic screening (*n* = 62), the most common risk factors that prompted screening were: maternal obesity (43.5%; 27/62), history of macrosomic baby (40.3%; 25/62), family history of DM (40.3%; 25/62), past history of GDM or impaired glucose tolerance (35.5%; 22/62) and intrauterine foetal death (33.9%; 21/62) (Figure 1).

### Factors associated with universal and risk-based screening

The bivariate analysis of factors associated with type of screening practice (universal vs risk-based screening) showed that provider gender, category and level of institution, job designation and cadre of doctors were each significantly associated with screening practice (*p* < 0.05) (Table 3). However, multivariate analysis showed no independent predictors of this screening practice.

**TABLE 3:** Factors associated with universal or risk-based screening using binary logistic regression analysis among antenatal healthcare providers in healthcare facilities in Jos North, Plateau state, Nigeria, August to September 2019.

Variable	Universal screening		Risk-based screening		Unadjusted *p*	Adjusted *p*
	*n*	%	*n*	%		
**Gender**					0.002	0.208
Male	33	60.0	22	40.0		
Female	54	85.2	9	14.3		
**Age group (years)**					0.111	0.376
20–29	28	87.5	4	17.5		
30–39	36	64.3	20	35.7		
40–49	18	75.0	6	25.0		
50–60	5	83.3	1	16.7		
**Hospital category**					0.038	0.364
Faith-based	6	50.0	6	50.0		
Private	48	82.8	10	17.2		
Government or public	33	68.8	15	31.1		
**Level of institution**					< 0.001	0.232
Primary	43	86.0	7	14.0		
Secondary	22	91.7	2	8.3		
Tertiary	22	50.0	22	50.0		
**Job designation**					< 0.001	0.205
Doctor	21	48.8	22	51.2		
Nurse or midwife	26	89.7	3	10.3		
Community health officer	5	55.6	4	44.4		
Community health extension worker	27	93.1	2	16.9		
Other	8	100.0	0	0.0		
**Care of doctor**					0.015	0.610
House officer	5	71.4	2	28.6		
Medical officer	8	77.7	3	27.3		
Registrar	0	0.0	4	100.0		
Senior registrar	1	14.3	6	85.2		
Consultant	3	30.0	7	70.0		
**Doctor’s specialty**					0.074	0.628
Family medicine	3	75.0	1	25.0		
Obstetrics and gynaecology	3	25.0	9	75.0		

### Method of testing

Among those who practised laboratory screening (*n* = 122), the most common glucose testing method was the glucose meter (glucometer) (88.5%; 108/122) (Table 2). This was followed closely by dipstick (82.0%; 100/122). Only 16/122 (13.1%) had automated chemistry analysers.

### Laboratory test used

**FIGURE 2:**
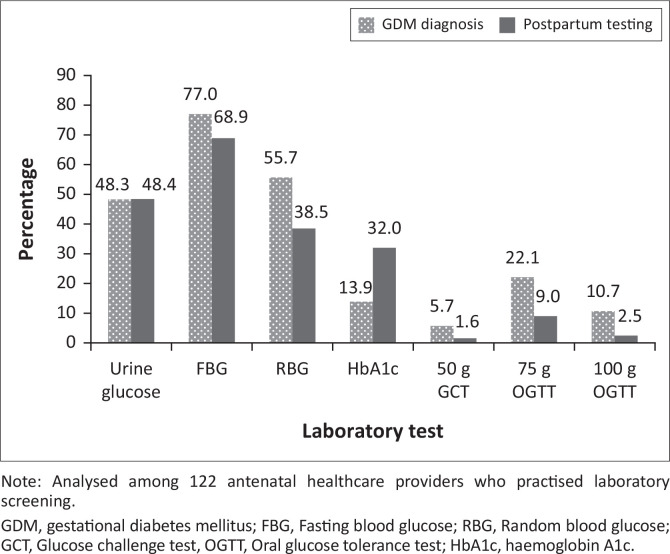
Laboratory testing for hyperglycaemia in pregnancy among antenatal healthcare providers in healthcare facilities in Jos North, Plateau state, Nigeria, August to September 2019.

Among those who practised laboratory screening (*n* = 122), the most commonly used test for diagnosing GDM was fasting blood glucose (FBG) (77.0%; 94/122), followed by random blood glucose (RBG) (55.7%; 68/122) (Figure 2). Very few used the recommended oral glucose tolerance test (OGTT): 22.1% (27/122) used the 75 g OGTT and 10.7% (13/122) used the 100 g OGTT. Postpartum glucose testing was mostly done using FBG (68.9%; 84/122).

**FIGURE 3:**
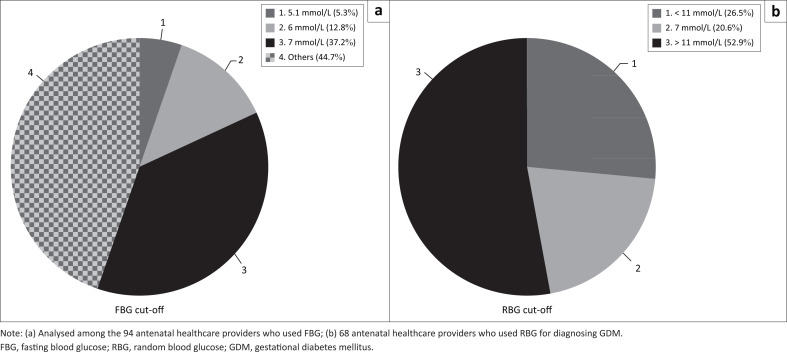
Threshold values of fasting blood glucose and random blood glucose for diagnosing gestational diabetes mellitus among antenatal healthcare providers in healthcare facilities in Jos North, Plateau state, Nigeria, August 2019 to September 2019. (a) fasting blood glucose threshold (b) random blood glucose.

Among the 94 AHPs who used FBG for diagnosing GDM, the most common threshold value cited was 7.0 mmol/L (37.2%; 35/94), 6.0 mmol/L (12.8%; 12/94) and 5.1 mmol/L (5.3%, 5/94) (Figure 3). There were 29 different cut-off values in all ranging from 2.5 mmol/L to 11.0 mmol/L. Among the 68 AHPs who used RBG for diagnosing GDM, there were 13 different threshold values ranging from 2.0 mmol/L to 15.0 mmol/L. Fourteen (20.6%; 14/68) AHPs mentioned a cut-off value of 11.0 mmol/L.

Further analysis (not illustrated) showed that only eight of the 27 AHP (29.6%) who used OGTT for diagnosing GDM mentioned the correct diagnostic thresholds for the three time points (0 h, 1 h and 2 h) for glucose measurement. Of the eight who used the correct diagnostic thresholds, seven aligned with the International Association of Diabetes and Pregnancy Study Groups criteria, while one aligned to the National Institute for Health and Care Excellence criteria. There were 20 different combinations of threshold values mentioned by the AHPs.

### Factors associated with diagnostic testing

The private and public hospitals (*p* = 0.014), primary and secondary healthcare facilities (*p* < 0.001), community health extension workers (*p* = 0.001), community health officers and nurses (*p* = 0.001) and facilities with available urine dipstick testing (*p* < 0.001) were more likely to use urine glucose for diagnosis of GDM (Table 4). Private institutions (*p* = 0.001) and facilities with available glucose meters (*p* = 0.005) were more likely to use FBG or RBG for diagnosing GDM.

**TABLE 4:** Factors associated with type of diagnostic test for gestational diabetes mellitus among antenatal healthcare providers in healthcare facilities in Jos North, Plateau state, Nigeria, August 2019 to September 2019.

Variable	Urine						FBG or RBG						OGTT					
	Yes		No		*X* ^2^	*p*	Yes		No		*X* ^2^	*p*	Yes		No		*X* ^2^	*p*
			
	*n*	%	*n*	%			*n*	%	*n*	%			*n*	%	*n*	%		
**Institution category**	-	-	-	-	8.75	0.014	-	-	-	-	15.26	0.001	-	-	-	-	13.35	0.001
Faith-based	1	8.3	11	91.7	-	-	9	75.0	3	25.0	-	-	8	66.7	4	33.3	-	-
Government or public	25	49.0	26	51.0	-	-	32	62.7	19	37.3	-	-	12	23.5	39	76.5	-	-
Private	32	54.2	27	45.8	-	-	55	93.2	4	6.8	-	-	10	16.9	49	83.1	-	-
**Level of care**	-	-	-	-	41.97	< 0.001	-	-	-	-	5.97	0.051	-	-	-	-	54.69	< 0.001
Primary	39	69.6	17	30.4	-	-	40	71.4	16	28.1	-	-	3	5.4	53	94.6	-	-
Secondary	16	66.7	8	33.3	-	-	23	95.8	1	4.2	-	-	0	0	24	100.0	-	-
Tertiary	3	7.1	39	92.9	-	-	33	78.6	9	21.4	-	-	27	64.3	15	35.7	-	-
**Job designation**	-	-	-	-	17.84	0.001	-	-	-	-	2.78	0.597	-	-	-	-	44.43	< 0.001
CHEW	19	67.9	9	32.1	-	-	19	67.9	9	32.1	-	-	1	3.6	27	96.4	-	-
CHO	5	55.6	4	44.4	-	-	7	77.8	2	22.2	-	-	1	11.1	8	88.9	-	-
Doctors	9	22.0	32	78.0	-	-	34	82.9	7	17.1	-	-	25	61.0	16	39.0	-	-
Nurses	21	60.0	14	40.4	-	-	29	82.9	6	17.1	-	-	2	5.7	33	94.3	-	-
Others	4	44.4	5	55.6	-	-	7	77.8	2	22.2	-	-	1	11.1	8	88.9	-	-
**Available glucometer**	-	-	-	-	2.28	0.162	-	-	-	-	7.76	0.005	-	-	-	-	0.001	0.970
Yes	54	50.0	1	50.0	-	-	89	82.4	19	12.5	-	-	26	24.1	82	75.9	-	-
No	4	28.6	53	50.0	-	-	7	50.0	7	50.0	-	-	4	28.6	10	71.4	-	-
**Available automated chemistry analysers**	-	-	-	-	1.96	0.162	-	-	-	-	0.89	0.356	-	-	-	-	0.001	0.011
Yes	5	31.3	11	68.8	-	-	14	87.5	2	12.5	-	-	8	50.0	8	50.0	-	-
No	53	50.0	53	50.0	-	-	82	77.4	24	22.6	-	-	22	20.8	84	79.2	-	-
**Available manual glucose**	-	-	-	-	0.005	0.944	-	-	-	-	0.016	0.898	-	-	-	-	0.001	0.989
Yes	1	50.0	1	50.0	-	-	1	50.0	1	50.0	-	-	1	50.0	1	50.0	-	-
No	57	47.5	63	52.5	-	-	95	79.2	25	20.8	-	-	29	24.2	91	75.8	-	-
**Available dipstick**	-	-	-	-	19.89	< 0.001	-	-	-	-	1.77	0.184	-	-	-	-	7.75	0.005
Yes	57	57.0	43	43.0	-	-	81	81.0	19	19.0	-	-	19	19.0	81	81.0	-	-
No	1	4.5	21	95.5	-	-	15	68.2	7	31.8	-	-	11	50.0	11	50.0	-	-

FBG, fasting blood glucose; RBG, random blood glucose; OGTT, oral glucose tolerance test; CHO, community health officer; CHEW, community health extension worker.

Antenatal healthcare providers in faith-based or government institutions (*p* = 0.001) and tertiary institutions (*p* < 0.001) were more likely to use OGTT for diagnosing GDM. Doctors compared to other categories of AHPs were more likely to use OGTT (*p* < 0.001). Antenatal healthcare providers in facilities with availability of automated glucose analyser were more likely to use OGTT (*p* = 0.011); however, AHPs in facilities where urine dipstick is used for glucose testing were less likely to use OGTT for diagnosing GDM (*p* = 0.005).

### Challenges to adequate practice

Late booking for antenatal care was cited as the most common challenge to screening and diagnosis of HIP identified by the respondents (85.2%; 104/122) (Table 2). This was followed by lack of guidelines (75.4%; 92/122), lack of information on GDM screening (63.9%; 78/122), lack of qualified personnel for testing (46.7%; 57/122) and lack of testing equipment (43.4%; 53/122).

## Discussion

The screening practice observed in this study showed that the majority of the respondents screen for GDM. Screening is carried out at all trimesters of pregnancy but most commonly during the second trimester. Thus, most AHPs follow the recommended practice of screening between 24 and 28 weeks. However, a large number of AHPs screen during first trimester. If this is done routinely for all pregnant women, it may reflect lack of knowledge or familiarity of recommended practice in screening guidelines. On the other hand, recent guidelines such as the World Health Organization and International Federation of Gynecology and Obstetrics, advise early screening for women with risk factors.^7,8^ Targeting screening at only 24–28 weeks might cause logistical challenges when considering that a substantial number of women in low-income countries may drop out or miss scheduled visits for various reasons such as lack of finance and disruption of services and poor accessibility to healthcare facilities, which is common in these settings.^15,16^ The high frequency of late screening for GDM observed in this study may be due to such factors, as uncertainty about gestational age and late presentation for antenatal care among Nigerian women is common, as reported by previous studies.^17,18,19,20^

In our study, almost 70% of the AHPs practised universal screening for HIP, whereas almost a quarter practised risk-based screening. This is similar to a survey of health workers in Morocco where more than two-thirds of the participants were in favour of universal screening and about one-third of providers preferred risk-based screening.^21^ In a Belgian survey, 83.9% of primary care physicians preferred universal screening.^22^ This finding suggests a trend toward universal screening. In a review article, Utz et al. published that as many as 80% of guidelines recommended universal screening.^12^ Universal screening for GDM provides broad coverage of screening for all pregnant women as part of comprehensive antenatal care. However, implementation of universal screening with the gold standard testing method, OGTT, may be problematic in low- and middle-income countries due to cost, equipment and staffing challenges, among others. This is amplified when considering that more than 95% of AHPs screened in our study used a laboratory method with or without an in-clinic method. Also, universal screening is the easier way out for this cadre of AHPs, who may lack the requisite knowledge and experience to sort pregnant women for screening based on risk factors.

Selective screening based on risk factors was practised by a quarter of the AHPs in our study, mostly doctors in clinical specialties or in tertiary settings. The majority of doctors in a survey in India preferred risk-based screening despite a national recommendation of universal screening.^17^ A previous study reported an established practice of risk-based screening in a teaching hospital in Ibadan, western Nigeria.^23^ This may reflect greater knowledge of selecting women at high risk for GDM using risk factors. The most common risk factors prompting screening for GDM in our study include maternal obesity, history of macrosomic babies, family history of DM, previous history of GDM or impaired glucose tolerance and intrauterine foetal death. This is in keeping with an earlier study in Jos, Nigeria, where the top four risk factors among women that were screened for GDM included history of macrosomic babies, maternal obesity, family history of DM and intrauterine foetal death.^24^

Selective screening based on risk factors is anchored on the need for cost-effectiveness, considering the high cost of screening all pregnant women. The critics of this approach have cited the fact that the prevalence of undiagnosed diabetes is rising among women of childbearing age and as much as 40% – 60% of women without the traditional risk factors for GDM may end up having GDM or overt diabetes in pregnancy and may be missed if only risk-based screening is adopted.^7,25^ In addition, failure to identify risk factors may occur due to gaps in historical recall of risk factors by patients, as well as poor, incomplete or unavailable medical records, which are common challenges in health systems in low- and middle-income countries.^12^ Furthermore, low compliance to risk-factor-based screening guidelines has been reported. As many as 70% of pregnant women with existing risk factors were not screened for GDM in a study in northern Sweden.^26^ The use of a structured, risk-factor checklist has been suggested to increase the effectiveness of identifying women with GDM in Ibadan, Nigeria.^23^

Glucometer and dipstick urine glucose testing were the most available testing methods in our study. This is not surprising considering the simplicity, affordability and availability of these testing methods. In our study, only a minority (13.1%) of the AHPs had access to automated chemistry analysers and, as expected, almost 70% of these were in tertiary care centres and none in primary healthcare settings. This finding underscores the claim that inability to test for glucose is a challenge for screening and management of HIP. The International Federation of Gynecology and Obstetrics has advocated the use of glucose meters in settings where it will be practically difficult to test with automated analysers.^7^ Bhavadharini et al. suggested that in settings where estimations by venous blood are not practicable, capillary blood glucose can be used as an initial screening test for GDM, using lower 2-h capillary blood glucose cut-off points to maximise the sensitivity.^27^ However, use of hand-held glucometers possesses some challenges, such as abuse by poorly trained personnel, need for calibration with a laboratory method, challenges with use of multiple glucometers from different vendors, quality control issues and the need for validation of current diagnostic criteria in different low- and middle-income country settings. Therefore, it is important that the validity and diagnostic performance of this method is explored in the Nigerian settings.

The availability of glucose meter and urine dipstick testing may also explain why many of the AHPs in our study adopted a universal screening strategy. While on the face of it, this is commendable, our results suggest that the majority of women were not screened according to the recommended screening method. The method for diagnosis of GDM preferred by AHPs in our study included urine glucose, FBG and RBG. These testing methods, although simple and readily available, lack sensitivity and run the risk of missing women with GDM. A similar finding was reported by Utz et al. in a Moroccan study where FBG was the most common test used by the respondents and urine glucose was commonly used by nurses.^21^ These methods also trade off diagnostic sensitivity compared to the gold standard method, OGTT. A previous study in Nigeria showed that as many as one in five women would be misclassified as normal glucose tolerant, if only FBG were used.^5^ Only a quarter of the AHPs in our study used the recommended diagnostic method of either 75 g or 100 g OGTT. The majority of these had good knowledge of HIP and practised in a tertiary care setting. On the other hand, practising in a primary care setting and availability of dipstick for glucose testing were associated with diagnosing GDM with urine glucose.

Although there was good agreement regarding the test used by AHPs in a given facility, there appeared to be some confusion with respect to the threshold used for making a diagnosis of GDM. Antenatal healthcare providers who worked within specific health facilities used different discriminatory cut-off values for diagnosing GDM. This is in agreement with previous studies in India by Utz et al. and Agarwal et al.^21,28^ The disparities in threshold for making a diagnosis of GDM portends grave danger of misclassification in managing women with HIP. Also, there are implications for referral of further care, if women are misclassified at lower cadre centres.

The post-partum follow-up for women with GDM is important for identifying sustained impaired glucose tolerance and those likely to progress to type 2 DM. It is recommended that pregnant women identified with GDM be tested 6–12 weeks post-partum.^14,29^ There are debates as to the most effective or suitable type of glucose testing during the post-partum period. Although a 75 g OGTT is widely recommended by major international guidelines, the use of RBG, FBG and glycated haemoglobin has been advocated in some quarters.^21^ It is not surprising though that, given the simplicity of FBG and RBG, these tests were the most preferred by AHPs in our study.

It is important to address identified challenges to adequate screening and diagnosis of GDM, if management of HIP is to be improved. The AHPs in our study were most concerned about late booking for antenatal care, which implies late testing for HIP. The consequence is that AHPs are afforded little time to intervene in cases of GDM or HIP in order to improve the pregnancy outcome. Three-quarters of the AHPs in our study agreed that issues concerning guidelines for screening and diagnosis of GDM, as well as general knowledge of HIP, present an arduous challenge to screening and diagnosis of HIP. It is commendable that in the last decade gains have been made towards harmonious international guidelines. It is therefore crucial that stakeholders in Nigeria work at developing a consensus for addressing the peculiarities of screening in the Nigerian context.

Lack of testing equipment and qualified personnel for testing were also identified as challenges to adequate screening and diagnosis of GDM in our study. It is important that laboratory personnel have the right testing methods and requisite knowledge to conduct the recommended OGTT for GDM. Glucose meters should be made widely available, but even then, it would be more appropriate that a 75 g OGTT be conducted with three glucose measurements than a once-off glucose measurement. This would require training of the AHPs at primary and secondary care levels.

### Recommendations

There is an urgent need for a harmonised screening and management guidelines for HIP that address the peculiar challenges of low-income settings. Lower cadre AHPs, especially those in primary healthcare settings, should be trained on standardised screening procedures for HIP like OGTT. The utility of hand-held glucose meters in identifying HIP and the impact of this in rural communities should be explored by stakeholders including obstetricians, endocrinologists and laboratorians. This should include studies to determine thresholds for local populations that will maximise the diagnostic capacity for identifying women with HIP using capillary blood glucose by available devices like glucose meters.

### Limitations

This study may have had some limitations. Because this is a cross-sectional study, it is difficult to establish temporal relationships. Also, the response of the AHPs on the issues concerning screening practices may be influenced by social desirability bias, although an effort was made to affirm the anonymous nature of the study. Some responses may have been influenced by the level of understanding of the operations in the facilities of employment. However, for such responses, only those who had worked for at least three months at the current facility were included in the analysis. Also, this study did not seek to elicit from the AHPs their reasons for their screening practices. Exploring these items would have made the number of questions too many which could have discouraged overall response. However, inferences were made from known factors that generally influence screening practices.

### Conclusion

In conclusion, this study found that the recommended testing methods are not available at lower levels of the healthcare system in Jos, North-Central Nigeria. Primary and secondary government or public health facilities lack even basic glucose testing devices like a glucose meter. The screening methods used for HIP generally involve simple testing methods like urine glucose tests and glucose meters, and the screening tests used do not generally conform to internationally recognised best practices.
